# Repurposing the Antibiotic D-Cycloserine for the Treatment of Hyperpigmentation: Therapeutic Potential and Mechanistic Insights

**DOI:** 10.3390/ijms26167721

**Published:** 2025-08-10

**Authors:** Ye-Jin Lee, Chang-Gu Hyun

**Affiliations:** Jeju Inside Agency and Cosmetic Science Center, Department of Chemistry and Cosmetics, Jeju National University, Jeju 63243, Republic of Korea; yyyyejin615@gmail.com

**Keywords:** B16F10, CREB, β-catenin, cosmeceutical, D-cycloserine, drug repurposing, hyperpigmentation, melanogenesis, MAPK pathway, PI3K/Akt pathway

## Abstract

Melanin overproduction contributes to hyperpigmentation disorders such as melasma and solar lentigines, leading to increasing demand for safe and effective skin-lightening agents. D-cycloserine (DCS), a known antimicrobial agent, has not been previously evaluated for dermatological applications. This study aimed to explore the potential of DCS as a novel anti-melanogenic compound and to elucidate its underlying molecular mechanisms in melanogenesis inhibition. The cytotoxicity and anti-melanogenic effects of DCS were assessed in B16F10 melanoma cells stimulated with α-MSH. Cell viability was determined via MTT assays, while melanin content, tyrosinase activity, and the expression levels of MITF, TYR, TRP-1, TRP-2, and major signaling proteins (e.g., CREB, MAPKs, GSK-3β/β-catenin) were evaluated using colorimetric assays and Western blotting. A 3D human skin model was also used to confirm in vitro findings, and a primary skin irritation test was conducted to assess dermal safety. DCS significantly reduced α-MSH-induced melanin content and tyrosinase activity without cytotoxicity at concentrations ≤100 µM. It downregulated MITF and melanogenic enzyme expression and modulated signaling pathways by enhancing ERK activation while inhibiting CREB, JNK, and p38 phosphorylation. Additionally, DCS suppressed β-catenin stabilization via GSK-3β activation. These effects were confirmed in a 3D human skin model, and a clinical skin irritation study revealed no adverse reactions in human volunteers. DCS exerts its anti-melanogenic effect by targeting multiple pathways, including CREB/MITF, MAPK, and GSK-3β/β-catenin signaling. Its efficacy and safety profiles support its potential as a novel cosmeceutical agent for the treatment of hyperpigmentation. Further clinical studies are warranted to confirm its therapeutic utility in human skin pigmentation disorders.

## 1. Introduction

Pigmentary disorders—ranging from hypopigmentary conditions such as vitiligo to hyperpigmentary states including melasma, post-inflammatory hyperpigmentation (PIH), and solar lentigines—affect hundreds of millions of individuals worldwide [1]. Although rarely life-threatening, these disorders impose considerable psychosocial morbidity and often persist despite current interventions such as phototherapy, topical corticosteroids, hydroquinone, and laser therapy, all of which are limited by relapse, variable efficacy, and long-term safety concerns [2,3]. Consequently, there is an unmet need for safe, affordable, and mechanistically novel treatments.

Drug repurposing—the systematic redeployment of approved or investigational agents for new indications—offers a cost- and time-efficient strategy for addressing such gaps [4]. Successful examples, including thalidomide for multiple myeloma, sildenafil for pulmonary arterial hypertension, and baricitinib for COVID-19, underscore the clinical and economic value of this approach [5,6,7].

D-cycloserine (DCS) is the R-enantiomer of 4-amino-1,2-isoxazolidin-3-one (C_3_H_6_N_2_O_2_), a natural antibiotic produced by *Streptomyces garyphalus*, *S. orchidaceus*, and *S. lavendulae* [8,9,10]. Clinically, DCS is used as a second-line agent for the treatment of multi-drug-resistant tuberculosis by inhibiting the peptidoglycan-synthesizing enzymes alanine racemase (ALR) and D-alanyl-D-alanine ligase (DDL) [11]. Interestingly, DCS also acts as a partial agonist at the glycine-binding site of N-methyl-D-aspartate (NMDA) receptors and has been investigated for the treatment of several neuropsychiatric disorders [12].

Beyond its antimicrobial and neuroactive properties, DCS has been suggested to possess immunomodulatory potential. Mechanistically, NMDA receptor-dependent calcium influx, oxidative stress, and mitogen-activated protein kinase (MAPK) signaling are closely associated with melanocyte biology and melanogenesis [13,14]. Recent studies have confirmed the presence of NMDA receptor-like channels in melanocytes and their contribution to intracellular Ca^2+^ dynamics and pigmentation signaling cascades [15,16].

Moreover, the NF-κB and MAPK pathways play central roles in cutaneous inflammatory signaling and are known to directly and indirectly influence pigmentation. Recent studies have shown that DCS suppresses NF-κB and MAPK activation in lipopolysaccharide (LPS)-stimulated RAW 264.7 macrophages [17], indicating its potential anti-inflammatory activity. Since inflammation-induced hyperpigmentation is a major concern in various skin disorders, the dual action of DCS on both neural and inflammatory pathways suggests its potential to modulate melanogenesis through mechanisms distinct from those of conventional depigmenting agents. Taken together, these findings support the hypothesis that DCS may regulate cutaneous pigmentation through alternative signaling pathways, allowing for the proposal of a novel mechanistic approach to pigmentation control.

The cosmetic sector has already capitalized on drug repurposing: azelaic acid (originally an anti-acne agent) is now a widely used skin-lightening ingredient [18,19]; tranexamic acid, a systemic antifibrinolytic, is exploited for melasma [20,21]; and dexpanthenol, once a wound-healing aid, is incorporated into moisturizing formulations for barrier repair [22,23]. Our group has similarly demonstrated that acenocoumarol, miglitol, imperatorin, and tobramycin possess previously unrecognized pigment-modulatory or anti-inflammatory properties, highlighting the breadth of cosmeceutical innovation achievable via repurposing [24,25,26,27].

Building on these insights, the present study investigated DCS as a prospective cosmeceutical agent for the treatment of pigmentary disorders. Specifically, we first quantified the effects of DCS on the expression of key melanogenic regulators—tyrosinase, tyrosinase-related protein-1 (TRP-1), TRP-2, and microphthalmia-associated transcription factor (MITF)—in cultured melanocytes [28]. To elucidate the underlying mechanisms, we examined the involvement of principal pigmentation-related signaling cascades, including the protein kinase A (PKA), p38 MAPK, and glycogen synthase kinase-3β (GSK-3β)/β-catenin pathways [29]. Finally, the dermatological safety of DCS was assessed via a primary human skin irritation test, thereby providing a comprehensive evaluation of its potential for topical application.

Through this multifaceted approach, we seek to provide a scientific foundation for repurposing an established antitubercular drug as a topical therapeutic for both hypo- and hyperpigmentation disorders, thereby broadening the translational scope and economic value of drug-repurposing strategies in dermatology.

## 2. Results and Discussion

### 2.1. DCS Inhibits Melanin Content and Tyrosinase Activity in B16F10 Cells

**Figure 1:**
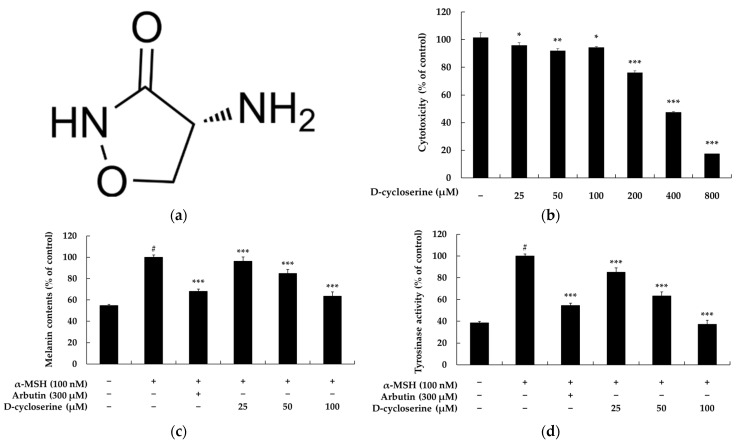
Effects of DCS on cell viability, melanin synthesis, and tyrosinase activity in B16F10 melanoma cells. (**a**) Chemical structure of DCS. (**b**) Cytotoxicity of DCS at concentrations ranging from 25 to 800 µM was assessed using the MTT assay after 72 h exposure. DCS showed dose-dependent cytotoxicity at concentrations ≥ 100 µM. (**c**) Melanin content was quantified in B16F10 cells stimulated with α-MSH (100 nM) and treated with DCS (25, 50, 100 µM) or arbutin (300 µM) for 72 h. DCS significantly reduced melanin content in a dose-dependent manner, comparable to arbutin. (**d**) Intracellular tyrosinase activity was measured under the same conditions as (**c**). DCS suppressed tyrosinase activity in a concentration-dependent fashion. Data are expressed as mean ± SD of three independent experiments. Statistical significance is denoted as # *p* < 0.001 compared to the untreated control group; * *p* < 0.05 and ** *p* < 0.01; and *** *p* < 0.001 compared to the α-MSH-treated group.

Identifying non-cytotoxic concentrations is a fundamental step in the preliminary safety assessment of newly developed bioactive ingredients. The MTT assay, a standard colorimetric method in cell viability testing, is extensively applied to evaluate the cytotoxic potential of pharmacological agents or chemicals. This assay relies on the enzymatic activity of NADH/NADPH-dependent cellular oxidoreductases, which catalyze the conversion of MTT into insoluble purple formazan crystals, indirectly reflecting cellular metabolic activity [30]. Thus, the MTT assay serves as an essential approach to defining a compound’s safety margin and selecting suitable dose ranges for further biological evaluations. Substances exhibiting cytotoxicity within specific dose ranges can be excluded from subsequent investigations, such as those targeting anti-melanogenic properties. Murine B16F10 melanoma cells are commonly employed as an in vitro model to study melanogenesis. These cells closely resemble normal human melanocytes in terms of melanin synthesis pathways, including key regulatory cascades such as cAMP/PKA, MAPK, and PI3K/AKT, and express melanogenic markers such as TRP-1, TRP-2, and the transcription factor MITF. Despite originating from mouse melanoma and thus having certain inherent limitations, B16F10 cells remain a valuable platform for exploring melanogenesis mechanisms and screening depigmenting agents for pharmaceutical or cosmetic applications [31,32]. To evaluate the cytotoxicity of DCS and determine its safe concentration range for functional assays, B16F10 cells were treated with increasing concentrations of DCS (25–800 μM) for 72 h, and cell viability was measured using the MTT assay. The results showed that DCS did not induce significant cytotoxicity at concentrations up to 100 μM, with cell viability exceeding 90%: 97% at 25 μM, 92% at 50 μM, and 94% at 100 μM, relative to untreated controls. However, exposure to higher concentrations led to marked reductions in viability: 77% at 200 μM, 47% at 400 μM, and 18% at 800 μM (Figure 1b). Based on these results, DCS concentrations of 25, 50, and 100 μM were selected for further analysis.

Subsequently, we examined the effect of DCS on melanogenesis in α-MSH-stimulated B16F10 cells. Treatment with α-MSH alone increased melanin content to 100%, compared to 54.61% in unstimulated control cells. Co-treatment with DCS resulted in a concentration-dependent decrease in melanin production: 3.81% at 25 μM, 11.22% at 50 μM, and 36.42% at 100 μM. These inhibitory effects were comparable to those of arbutin (32.07% at 300 μM), a well-known depigmenting agent (Figure 1c). In addition, DCS significantly suppressed tyrosinase activity, which was elevated by α-MSH stimulation. Co-treatment with DCS reduced tyrosinase activity to 14.91%, 36.82%, and 62.94% at 25, 50, and 100 μM, respectively, compared to 100% in the α-MSH-treated group. Arbutin treatment also lowered tyrosinase activity to 45.56%, supporting the anti-melanogenic potential of DCS (Figure 1d). Collectively, these findings indicate that DCS effectively inhibits melanogenesis by reducing both melanin content and tyrosinase activity, without inducing cytotoxicity at concentrations up to 100 μM.

### 2.2. DCS Regulates the Expression of Melanogenesis-Related Proteins in B16F10 Cells

**Figure 2:**
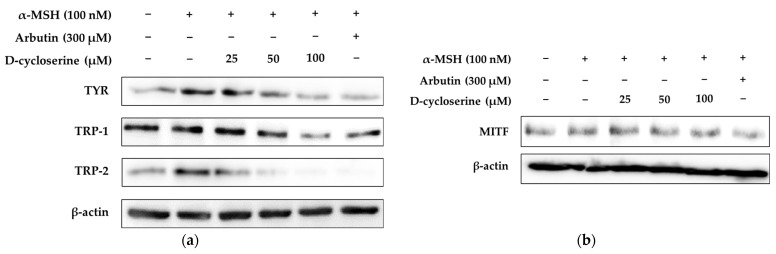

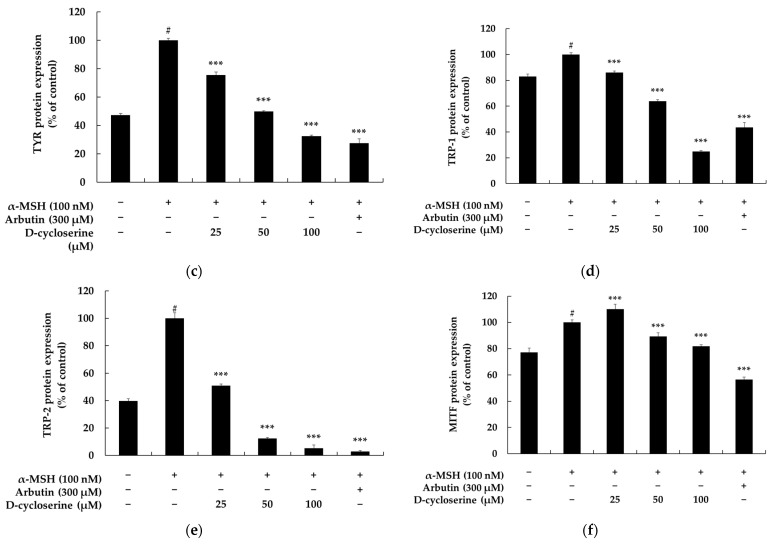
Effects of DCS on melanogenic protein and MITF expression in α-MSH-stimulated B16F10 cells. (**a**,**b**) Representative Western blot images showing the expression levels of TYR, TRP-1, TRP-2, and MITF in B16F10 cells treated with α-MSH (100 nM), DCS (25, 50, or 100 µM), or arbutin (300 µM) for 48 h. β-actin was used as the internal loading control. (**b**–**d**) Quantitative densitometric analysis of TYR (**c**), TRP-1 (**d**), TRP-2 (**e**), and MITF (**f**) protein levels relative to β-actin, expressed as percentage of the untreated control. α-MSH stimulation significantly upregulated the expression of melanogenic proteins, whereas treatment with DCS reduced their expression in a dose-dependent manner, similar to the effect of arbutin. Protein band intensities were quantified using ImageJ software (version 9.4.0), normalized to β-actin, and expressed as the mean ± SD from at least three independent experiments. Statistical significance is denoted as # *p* < 0.001 compared to the untreated control group and *** *p* < 0.001 compared to the α-MSH-treated group.

Melanogenesis, the process of melanin pigment synthesis within melanocytes, is a crucial physiological process that protects the skin from ultraviolet radiation and determines skin color. This process is precisely regulated by specialized enzymes and transcription factors within the melanocytes. Key proteins that play pivotal roles in this pathway include tyrosinase (TYR), TRP-1, TRP-2, and MITF [28]. TYR, serving as the rate-limiting enzyme in melanin biosynthesis, catalyzes the oxidation of tyrosine to DOPA (L-3,4-dihydroxyphenylalanine) and subsequently to DOPAquinone. This enzyme plays a decisive role in the initial stages of melanin production, and most depigmenting agents exert their effects by directly inhibiting its activity [33]. TRP-1 is an oxidoreductase enzyme that converts DOPAchrome to DHICA (5,6-dihydroxyindole-2-carboxylic acid), influencing the quality of melanin (distinguishing between black/brown eumelanin). TRP-1 also contributes to regulating the stability and functional activity of TYR. TRP-2 (also known as DOPAchrome tautomerase) catalyzes the isomerization of DOPAchrome to DHICA, thereby regulating the latter part of the melanogenesis pathway. TRP-2 can also influence the structural characteristics and antioxidant capacity of melanin [28,33]. The expression of these melanogenic enzymes is transcriptionally regulated upstream by MITF. MITF directly binds to the promoter regions of the TYR, TRP-1, and TRP-2 genes, thereby inducing their transcription. Consequently, MITF is regarded as the ‘master regulator’ that orchestrates melanocyte differentiation and survival, and melanin synthesis, making the modulation of its expression a widely studied target for controlling melanogenesis [34,35]. In conclusion, TYR, TRP-1, TRP-2, and MITF, as essential components of melanogenesis, represent major targets for the development of skin-whitening agents. Strategies aimed at inhibiting their activity or suppressing their expression are considered promising approaches for the treatment and prevention of hyperpigmentary skin disorders such as melasma, freckles, and solar lentigines. Recent research also actively explores the development of multi-targeting compounds that consider upstream signaling pathways regulating these proteins, offering a comprehensive approach to melanin modulation [36,37,38]. To investigate the effect of DCS on melanogenesis-associated proteins, we evaluated the protein expression levels of MITF, TYR, and tyrosinase-related proteins TRP-1 and TRP-2 in α-MSH-stimulated melanocytes. As shown in the Western blot and quantified bar graphs, α-MSH significantly increased the expression of MITF, TYR, TRP-1, and TRP-2 compared to the untreated control group, indicating activation of melanogenic pathways. Treatment with 300 µM arbutin, a known depigmenting agent, effectively attenuated this α-MSH-induced upregulation, serving as a positive control. DCS treatment resulted in a concentration-dependent suppression of MITF protein expression, with the most notable reduction observed at 100 µM, decreasing MITF expression to approximately 43.56% of the α-MSH-stimulated level (*p* < 0.001). This downregulation of MITF was associated with a concomitant reduction in its downstream targets TYR, TRP-1, and TRP-2. TYR expression was significantly decreased to 34.53%, 50.11%, and 67.43% at 25, 50, and 100 µM DCS, respectively (*p* < 0.001). TRP-1 expression followed a similar trend, decreasing to 13.91%, 36.06%, and 75.14%, while TRP-2 expression was drastically suppressed to 49.07%, 87.63%, and 94.65% at increasing concentrations of DCS (*p* < 0.001 in all cases). These findings indicate that DCS exerts its anti-melanogenic effect by targeting the MITF signaling axis, resulting in the downregulation of key melanogenic enzymes (Figure 2). The consistent inhibition of MITF and its downstream targets supports the potential of DCS as a promising candidate for skin-whitening or anti-hyperpigmentation therapeutics.

### 2.3. DCS Inhibits Melanogenesis Through the GSK–3β/β-Catenin Pathway in B16F10 Cells

Melanogenesis is a complex biological process that determines skin pigmentation and provides photoprotection against ultraviolet radiation. Among the multiple signaling cascades regulating this process, the GSK-3β/β-catenin pathway has emerged as a critical regulatory axis. GSK-3β, a serine/threonine kinase, phosphorylates β-catenin, targeting it for proteasomal degradation. When GSK-3β is inhibited—either by upstream signals such as α-melanocyte-stimulating hormone (α-MSH), Wnt signaling, or the PI3K/AKT pathway—β-catenin escapes degradation, accumulates in the cytoplasm, and translocates to the nucleus. There, it forms transcriptional complexes with TCF/LEF transcription factors and promotes the expression of MITF, a master regulator of melanogenesis. Upregulated MITF enhances the transcription of key melanogenic enzymes including tyrosinase, TRP-1, and TRP-2, thereby promoting melanin synthesis. Conversely, sustained or enhanced GSK-3β activity leads to increased degradation of β-catenin, resulting in reduced MITF transcription and subsequent downregulation of melanin production. Given the central role of the GSK-3β/β-catenin axis in melanogenesis, this pathway represents an attractive pharmacological target for the development of skin-lightening agents [39,40,41]. Indeed, a variety of natural products and synthetic compounds have been reported to inhibit melanin synthesis by modulating this pathway. Therefore, fine-tuning the GSK-3β/β-catenin signaling cascade holds significant promise as a strategic approach for developing safe and effective treatments for hyperpigmentation disorders and cosmetic skin whitening [42,43,44].

**Figure 3:**
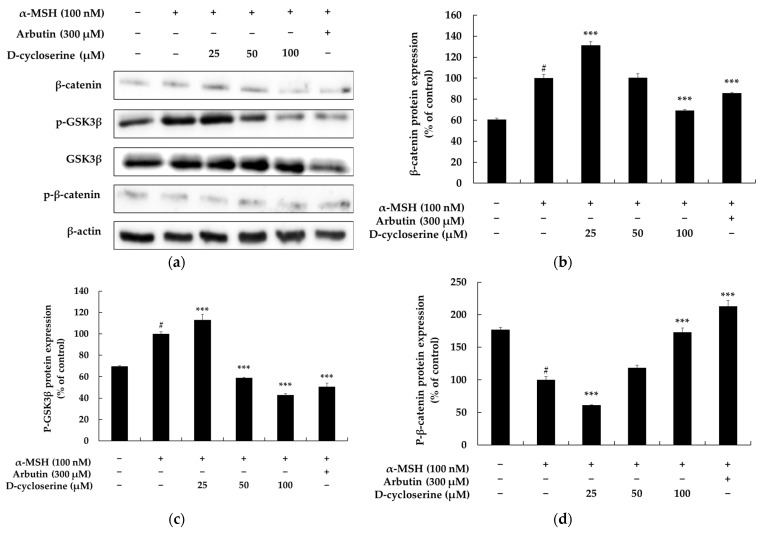
Effects of DCS on the GSK-3β/β-catenin signaling pathway in α-MSH-stimulated B16F10 cells. (**a**) Representative Western blot images showing the expression of total β-catenin, total and phosphorylated GSK-3β (p-GSK-3β), and phosphorylated β-catenin (p-β-catenin) following treatment with α-MSH (100 nM), DCS (25, 50, or 100 µM), or arbutin (300 µM). β-actin was used as the internal loading control. (**b**–**d**) Results of densitometric analysis of total β-catenin (**b**), p-GSK-3β (**c**), and p-β-catenin (**d**) expression levels, normalized to β-actin and expressed as percentage of the untreated control. Treatment with α-MSH increased β-catenin and p-GSK-3β levels while reducing p-β-catenin, consistent with the activation of melanogenesis. DCS reversed these effects in a dose-dependent manner, indicating that it promotes β-catenin degradation and suppresses melanogenic signaling through GSK-3β activation. Protein band intensities were quantified using ImageJ software and normalized to β-actin and are expressed as the mean ± SD from at least three independent experiments. Statistical significance is indicated as # *p* < 0.001 compared to the untreated control group and *** *p* < 0.001 compared to the α-MSH-treated group.

To explore the involvement of the GSK-3β/β-catenin signaling pathway in the anti-melanogenic activity of DCS, we assessed the protein levels of β-catenin, phosphorylated GSK-3β (p-GSK3β), and phosphorylated β-catenin (p-β-catenin) in α-MSH-stimulated melanocytes using Western blot analysis (Figure 3a). As shown in Figure 3b, treatment with α-MSH (100 nM) significantly increased β-catenin protein expression to 100%, compared to 60.60% in the untreated control group (*p* < 0.05), indicating a 39.40% elevation. This suggests that α-MSH stabilizes β-catenin by inhibiting its proteasomal degradation. However, co-treatment with α-MSH and 300 μM arbutin reduced β-catenin expression to 85.65%, representing a 14.35% decrease compared to α-MSH alone, which is consistent with the known anti-melanogenic action of arbutin via inhibition of β-catenin accumulation.

Treatment with DCS induced a biphasic effect on β-catenin expression. At 25 μM, DCS significantly increased expression to 131.34% (*p* < 0.001 vs. α-MSH), while 50 μM DCS maintained expression at 100.42%, comparable to α-MSH alone. However, 100 μM DCS led to a decrease in β-catenin levels to 69.13%, a 30.87% reduction, suggesting that higher concentrations of DCS may promote β-catenin degradation.

To explore the upstream regulation of β-catenin stability, we analyzed the phosphorylation level of GSK-3β (Figure 3c). α-MSH increased p-GSK3β expression from a basal level of 69.91% to 100%, indicating inhibition of GSK-3β activity. In contrast, co-treatment with arbutin and α-MSH reduced p-GSK3β levels to 50.64%, corresponding to a 49.36% reduction. DCS co-treatment resulted in dose-dependent decreases in p-GSK3β expression to 113.12% (25 μM), 59.99% (50 μM), and 42.93% (100 μM), all statistically significant compared to α-MSH alone (*p* < 0.001). These data suggest that DCS restores GSK-3β activity by suppressing its inhibitory phosphorylation, thereby promoting β-catenin degradation.

Consistent with this, Figure 3d shows that p-β-catenin levels were inversely modulated. α-MSH treatment reduced p-β-catenin from a basal level of 176.85% to 100% (*p* < 0.05), indicating stabilization of β-catenin. DCS restored p-β-catenin expression in a dose-dependent manner to 61.04%, 118.78%, and 172.80% at 25, 50, and 100 μM, respectively (*p* < 0.001). Co-treatment with arbutin also elevated p-β-catenin to 213.12%, further supporting enhanced β-catenin degradation.

Notably, the apparent paradox at 100 μM DCS—where p-β-catenin is elevated while total β-catenin is decreased—can be mechanistically explained by GSK-3β-mediated proteasomal degradation. As 100 μM DCS markedly reduces p-GSK3β, GSK-3β is reactivated and promotes the phosphorylation of β-catenin, marking it for ubiquitin-dependent proteasomal degradation. This process explains the simultaneous increase in p-β-catenin and decrease in total β-catenin levels and highlights the role of DCS in accelerating β-catenin turnover.

Taken together, these results demonstrate that DCS suppresses melanogenesis through regulation of the GSK-3β/β-catenin signaling pathway. DCS promotes GSK-3β activity by reducing p-GSK3β levels, leading to increased phosphorylation and subsequent proteasomal degradation of β-catenin. This ultimately attenuates MITF transcription and expression of melanogenic enzymes.

### 2.4. DCS Inhibits Melanogenesis Independently of the PI3K/Akt Pathway in B16F10 Cells

**Figure 4:**
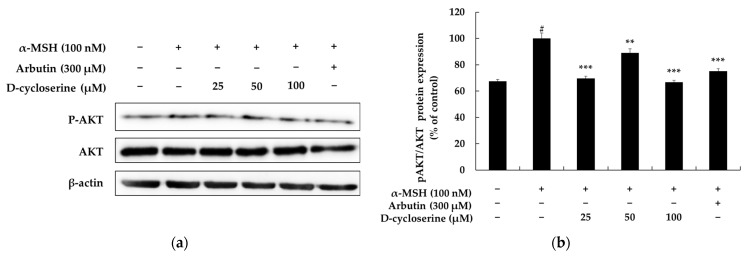
Effect of DCS on AKT signaling pathway protein expression in α-MSH-stimulated B16F10 cells. (**a**) Representative Western blot images showing the protein levels of phosphorylated AKT (p-AKT) and total AKT following treatment with α-MSH (100 nM), DCS (25, 50, or 100 µM), or arbutin (300 µM). β-actin was used as the internal control. (**b**) Results of densitometric analysis of p-AKT/AKT protein expression ratio, normalized to β-actin and expressed as a percentage of the untreated control. α-MSH stimulation significantly increased AKT phosphorylation, while DCS dose-dependently reduced p-AKT levels, suggesting that DCS suppresses the melanogenic PI3K/AKT pathway. Data represent the mean ± SD from at least three independent experiments. ** *p* < 0.01, *** *p* < 0.001 vs. α-MSH-only group; # *p* < 0.001 vs. untreated control.

The PI3K/AKT (phosphatidylinositol 3-kinase/protein kinase B) signaling pathway is a crucial regulator of melanogenesis; it is involved in various molecular mechanisms related to melanocyte survival, proliferation, differentiation, and pigment synthesis. This pathway is activated by diverse external stimuli, including the stimulation of the melanocortin 1 receptor (MC1R) by α-melanocyte-stimulating hormone (α-MSH). It plays a vital role in regulating the stability and activity of MITF, a key transcription factor essential for melanin production. Upon activation of PI3K, AKT becomes phosphorylated and activated, a process mediated by the formation of PIP3 (phosphatidylinositol-3,4,5-trisphosphate). Activated AKT, in turn, phosphorylates and thereby inactivates GSK-3β. Consequently, this prevents the degradation of β-catenin, leading to its nuclear accumulation. As β-catenin contributes to the transcriptional activation of MITF, the activation of the PI3K/AKT pathway is linked to the promotion of melanin synthesis. Furthermore, AKT directly influences the stability of MITF itself, thereby regulating the expression of melanogenesis-related genes such as tyrosinase, TRP-1, and TRP-2. Conversely, inhibition of PI3K/AKT signaling leads to reduced MITF expression, decreased activity of melanogenic enzymes, and, ultimately, suppression of pigment production. In conclusion, the PI3K/AKT signaling pathway is an important pro-melanogenic pathway. Strategies aimed at selectively inhibiting this pathway represent a highly promising approach for the development of functional cosmetics or therapeutic agents for skin whitening and the alleviation of hyperpigmentation [43,44,45,46,47]. To investigate the potential involvement of the AKT signaling pathway in DCS-mediated effects on melanogenesis, we evaluated the phosphorylation status of AKT in melanocytes under various treatment conditions. As shown in Figure 4, basal pAKT/AKT protein expression in untreated control cells was approximately 67.41%. Stimulation with 100 nM α-MSH, a known activator of melanogenesis, significantly increased pAKT/AKT expression to 100% (Figure 4), confirming successful activation of the AKT pathway. Treatment with 300 μM arbutin, used as a positive control, significantly inhibited pAKT/AKT expression in the presence of α-MSH (24.78%). However, a notable suppression of α-MSH-induced AKT phosphorylation was observed when melanocytes were co-treated with α-MSH and varying concentrations of DCS. Specifically, 25 μM DCS inhibited pAKT/AKT expression by 30.36%, while 50 μM and 100 μM inhibited it by 10.87% and 33.17%, respectively (Figure 4). These results indicate that DCS effectively inhibits the α-MSH-induced activation of the AKT signaling pathway, suggesting its potential role in modulating melanogenesis through this mechanism. In parallel, our data also showed that DCS treatment led to a dose-dependent decrease in the phosphorylated (inactive) form of GSK-3β at Ser9 (Figure 3), indicating GSK-3β activation. As AKT is a known upstream negative regulator of GSK-3β, the observed activation of GSK-3β is consistent with the inhibition of PI3K/AKT signaling. This suggests that DCS does not independently activate GSK-3β, but rather promotes its activation via upstream inhibition of the AKT pathway. These findings collectively support a mechanistic link in which DCS inhibits the PI3K/AKT axis, thereby relieving the inhibitory phosphorylation of GSK-3β, leading to its activation and contributing to downstream effects on melanogenesis.

### 2.5. DCS Inhibits Melanogenesis Through the MAPK Pathway in B16F10 Cells

**Figure 5:**
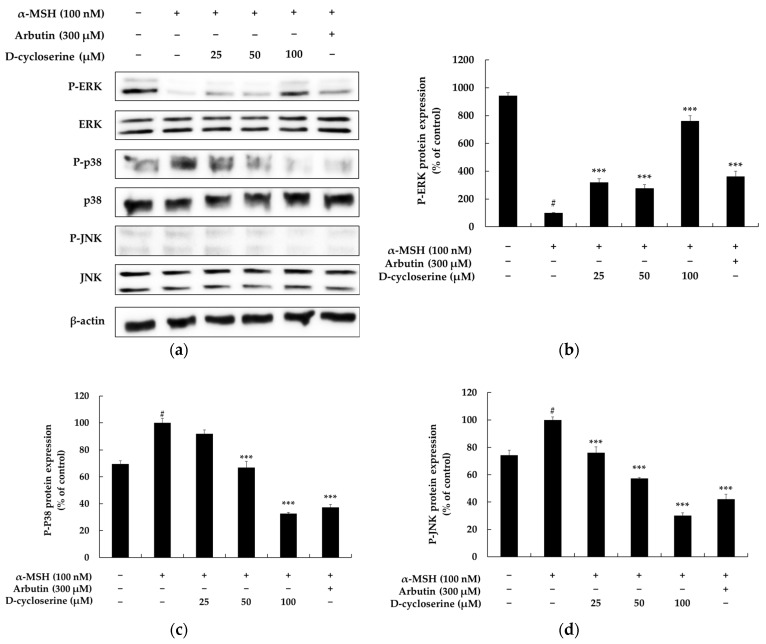
Effects of DCS on MAPK signaling pathways in α-MSH-stimulated B16F10 melanoma cells. (**a**) Representative Western blot images showing the phosphorylation and total protein levels of ERK, p38, and JNK following treatment with α-MSH (100 nM), DCS (25, 50, or 100 µM), or arbutin (300 µM). β-actin was used as the loading control. (**b**–**d**) Results of densitometric analyses of phosphorylated ERK (**b**), p38 (**c**), and JNK (**d**) protein levels, normalized to their respective total proteins and β-actin, and expressed as percentages of the control. α-MSH stimulation markedly increased the phosphorylation of all three MAPKs. DCS treatment significantly downregulated the phosphorylation of p38 and JNK in a dose-dependent manner and partially attenuated ERK activation. Statistical significance is indicated as # *p* < 0.001 compared to the untreated control group and *** *p* < 0.001 compared to the α-MSH-treated group.

The MAPK signaling pathway plays a crucial role in regulating melanin biosynthesis. It is one of the main upstream signaling pathways that directly controls the expression and activity of MITF, which functions as a key regulator in the process of melanogenesis. The MAPK pathway is broadly divided into three major subfamilies, extracellular signal-regulated kinase (ERK), c-Jun N-terminal kinase (JNK), and p38 MAPK, with each modulating melanin synthesis through distinct mechanisms. For instance, the ERK pathway generally tends to inhibit melanogenesis by inducing MITF degradation. When ERK is activated, MITF undergoes phosphorylation, which subsequently promotes its ubiquitination and proteasomal degradation, leading to a decrease in its protein levels. This reduction, in turn, suppresses the expression of key melanogenic enzymes such as tyrosinase, TRP-1, and TRP-2, thereby inhibiting melanin production. Conversely, the p38 MAPK pathway generally tends to promote melanin biosynthesis by increasing the transcriptional activity of MITF. Activation of p38 MAPK stabilizes MITF and induces the expression of various melanogenic enzyme genes, ultimately contributing to increased pigmentation. The JNK pathway also participates in the survival and function of melanocytes. It can be activated in response to stress signals or ultraviolet (UV) exposure, consequently influencing melanogenesis. Its precise role can be context-dependent, exhibiting either inhibitory or stimulatory effects depending on the cellular environment and specific stimuli. The clear duality and specialized functions of these MAPK pathways render them highly significant targets for the development of treatments for pigmentary disorders. For example, inhibitors of p38 or JNK could be utilized as effective skin-whitening or anti-hyperpigmentation therapeutic agents by suppressing excessive melanin synthesis. Conversely, activators of the ERK pathway could induce a similar depigmenting effect by promoting MITF degradation [48,49]. Indeed, numerous natural products and small molecules have demonstrated anti-pigmentary activity by targeting these pathways, and a considerable number of candidate compounds for treating pigmentary disorders based on MAPK signaling modulation have been reported. In conclusion, the MAPK pathway serves as a central regulatory axis of melanogenesis, with each subfamily capable of either promoting or inhibiting melanin production depending on its specific context. Consequently, modulating MAPK activity represents an effective pharmacological target for treating various pigmentary skin disorders, including melasma, freckles, and solar lentigines. In this regard, the discovery and mechanistic investigation of compounds that selectively modulate MAPK signaling are considered crucial strategies for the development of new drugs for skin whitening and the treatment of pigmentary disorders [50,51,52]. To investigate the involvement of major MAPK signaling pathways in DCS-mediated anti-melanogenic effects, we examined the phosphorylation levels of ERK, p38, and JNK in α-MSH-stimulated melanocytes via Western blotting (Figure 5a). As shown in Figure 5b, treatment with 100 nM α-MSH markedly suppressed ERK phosphorylation, reducing p-ERK protein expression by approximately 89.38% compared to untreated control cells (from 942.67% to 100%). However, co-treatment with DCS significantly restored this suppression in a dose-dependent manner. Specifically, p-ERK expression increased by 276.20%, 319.36%, and 761.50% at 25 µM, 50 µM, and 100 µM DCS, respectively, relative to the α-MSH-only group (*p* < 0.001). Similarly, 300 µM arbutin co-treatment increased p-ERK levels by 362.60% compared to α-MSH alone (*p* < 0.001), confirming the reversal of α-MSH-induced ERK inhibition by both agents. In contrast, α-MSH stimulation significantly enhanced p38 phosphorylation, increasing p-p38 expression by 30.55% compared to the control (from 69.45% to 100%). Co-treatment with DCS effectively inhibited this elevation in a dose-dependent manner. At 25 µM, 50 µM, and 100 µM DCS, p-p38 expression decreased by 8.23%, 33.18%, and 76.42%, respectively, relative to α-MSH alone (*p* < 0.001). Arbutin co-treatment also reduced p-p38 levels by 62.68% (*p* < 0.001), indicating that DCS and arbutin suppress α-MSH-induced activation of the p38 MAPK pathway. Similarly, α-MSH increased p-JNK protein expression by 25.70% compared to the untreated control group. Co-treatment with DCS resulted in a dose-dependent reduction in p-JNK levels: 23.92%, 42.67%, and 69.87% lower than α-MSH alone at 25 µM, 50 µM, and 100 µM DCS, respectively (*p* < 0.001). Arbutin co-treatment also inhibited p-JNK expression by 57.90% compared to α-MSH treatment (*p* < 0.001). These findings suggest that DCS exerts its anti-melanogenic effects via dual modulation of MAPK signaling—by upregulating ERK phosphorylation and concurrently inhibiting the stress-activated kinases p38 and JNK—thereby mimicking the molecular action profile of known depigmenting agents like arbutin. However, since the phosphorylation of these signaling molecules was evaluated at a single fixed time point (6 h), the temporal dynamics of pathway activation remain unclear. We acknowledge this as a limitation, and future studies incorporating time-course analyses (e.g., at 0.5, 1, 3, and 3 h) will be necessary to clarify the kinetics and sequence of DCS-mediated signaling events.

### 2.6. DCS Inhibits α-MSH-Induced CREB Phosphorylation in Melanocytes

**Figure 6:**
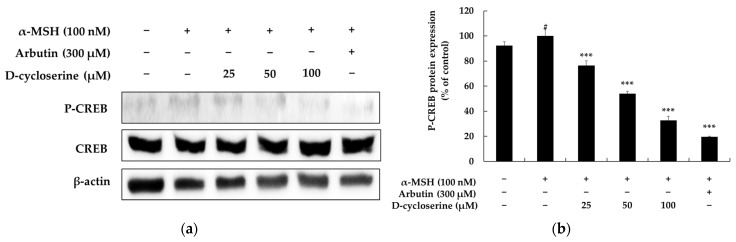
Effects of DCS on CREB phosphorylation in α-MSH-stimulated B16F10 melanoma cells. (**a**) Representative Western blot images showing levels of phosphorylated CREB (p-CREB) and total CREB after treatment with α-MSH (100 nM), DCS (25, 50, or 100 µM), or arbutin (300 µM). β-actin was used as a loading control. (**b**) Results of densitometric analysis of p-CREB protein levels, normalized to total CREB and β-actin, and expressed as percentages relative to the control group. α-MSH markedly enhanced p-CREB expression, while DCS treatment significantly and dose-dependently suppressed CREB phosphorylation. Statistical significance is indicated as # *p* < 0.001 compared to the untreated control group and *** *p* < 0.001 compared to the α-MSH-treated group.

CREB, a cAMP response element-binding protein, is a pivotal downstream effector of the cAMP/PKA cascade—the principal upstream regulator of melanogenesis that is activated by diverse stimuli such as ultraviolet radiation. When α-MSH binds to its cognate melanocortin-1 receptor (MC1R), intracellular cAMP rises, PKA becomes activated, and PKA phosphorylates CREB at Ser133 to generate phospho-CREB (p-CREB). The latter translocates to the nucleus, binds cAMP response elements (CRE) in genomic DNA, and drives transcription of MITF, which in turn up-regulates the melanogenic enzymes tyrosinase, TRP-1 and TRP-2, ultimately heightening melanin synthesis; thus, modulation of CREB is recognized as a strategic therapeutic entry point for treating pigmentary disorders [53,54,55]. In this context, we examined how DCS influences CREB phosphorylation. Baseline p-CREB expression in untreated B16F10 cells was approximately 90%, and treatment with 100 nM α-MSH increased p-CREB to 100%, confirming activation of the cAMP/PKA/CREB axis. However, co-treatment with DCS markedly suppressed α-MSH-induced CREB phosphorylation in a dose-dependent manner. Compared to α-MSH alone, 25 µM DCS reduced p-CREB by 27.68%, 50 µM by 48.24%, and 100 µM by 69.83% (all *p* < 0.001). Co-treatment with 300 µM arbutin also significantly decreased p-CREB levels by 81.29% (*p* < 0.001 vs. α-MSH group) (Figure 6). These results indicate that DCS strongly attenuates α-MSH-induced activation of the PKA/CREB pathway, suggesting that inhibition of CREB phosphorylation may be a key mechanism by which DCS exerts its anti-melanogenic effects.

### 2.7. Cutaneous Safety Evaluation of DCS

A standard primary skin irritation patch test was conducted on 32 healthy Korean adults (31 females and 1 male) aged 24 to 55 years (mean ± SD: 46.6 ± 8.2 years). A single occlusive patch containing the test material (DCS) was applied to the upper back area for 24 h. Dermal reactions were evaluated at 20 min and again at 24 h after patch removal, based on standardized scoring criteria for erythema and edema. All subjects received a score of “zero” at both time points, indicating no signs of irritation. No protocol deviations, dropouts, or adverse events occurred during the test (Table 1). These results demonstrate that DCS is well tolerated by human skin under the conditions tested, supporting its suitability for topical application.

All participants in the irritation study were of East Asian descent (Korean), and based on inclusion criteria and clinical observation, their Fitzpatrick skin types were estimated to fall between types III and IV. While this phototype range reflects the typical characteristics of the local population, it limits the generalizability of the findings to individuals with darker skin types (Fitzpatrick types V–VI), who may exhibit distinct cutaneous responses due to higher baseline melanogenic activity and variations in skin barrier function. To address this limitation, future studies should be designed to include more ethnically and phototypically diverse populations. Such expanded clinical evaluations will be essential to fully assess the cutaneous safety and efficacy of DCS across global skin types, and to support its broader applicability as a cosmeceutical agent.

## 3. Materials and Methods

### 3.1. Chemicals and Reagents

D-cycloserine (C6880), arbutin (A4256), α-melanocyte-stimulating hormone (α-MSH, M4135), 3-(4,5-dimethylthiazol-2-yl)-2,5-diphenyltetrazolium bromide (MTT, M5655), protease inhibitor cocktail (P8340), and 2-mercaptoethanol (60-24-2) were purchased from Sigma-Aldrich (St. Louis, MO, USA). Dulbecco’s Modified Eagle’s Medium (DMEM, 11995073) and penicillin-streptomycin (P/S, 15140122) were obtained from Gibco (Waltham, MA, USA), and fetal bovine serum (FBS, 30-2020) was sourced from ATCC (Manassas, VA, USA). The radioimmunoprecipitation assay (RIPA) buffer (R2002) was supplied by BioSesang (Seongnam, Republic of Korea), and the bicinchoninic acid (BCA) protein assay kit (712853) was from MilliporeSigma (Burlington, MA, USA). Laemmli sample buffer (161-0737) was obtained from Bio-Rad, and blocking agents including skim milk (232100) and bovine serum albumin (BSA100-E) were purchased from BD Difco (Franklin Lakes, NJ, USA) and Bovogen (Keilor East, Australia), respectively.

Primary antibodies against tyrosinase (SC-20035), TRP-1 (SC-166857), TRP-2 (SC-74439), MITF (SC-71588), and β-actin (SC-47778) were obtained from Santa Cruz Biotechnology (Dallas, TX, USA). Antibodies targeting phospho- and total forms of β-catenin (9561S, 25362S), GSK-3β (9322S, 5676S), CREB (9198S, 4820S), ERK (9102S, 9101S), p38 (9211S, 9212S), JNK (9251S, 9252S), and AKT (9271S, 9272S) were purchased from Cell Signaling Technology (Danvers, MA, USA). HRP-conjugated anti-rabbit IgG (7074S) and anti-mouse IgG (7076S) secondary antibodies were also from Cell Signaling Technology.

The instruments used in this study included a shaker incubator (MB100-4A, Thermo Fisher Scientific, Waltham, MA, USA), a refrigerated centrifuge (M15R, Hanil Scientific), a microplate spectrophotometer (Epoch, BioTek), a trans-blot turbo transfer system (Trans-Blot, Bio-Rad), a power supply (PowerPac Basic, Bio-Rad), and an imaging system (FUSION Solo S, Vilber Lourmat).

### 3.2. Cell Culture

Murine melanoma B16F10 cells (ATCC CRL-6475) were cultured in Dulbecco’s Modified Eagle’s Medium (DMEM) supplemented with 10% (*v*/*v*) heat-inactivated fetal bovine serum (FBS) and 1% (*v*/*v*) penicillin-streptomycin (P/S). Cells were maintained at 37 °C in a humidified incubator with 5% CO_2_. Subculturing was performed when the cells reached 70–80% confluence, and cells between passages 5 and 20 were used for all experiments.

### 3.3. Cell-Viability (MTT) Assay

B16F10 cells were seeded into 24-well plates at 1.5 × 10^4^ cells well^−1^ and allowed to attach for 24 h at 37 °C in a humidified atmosphere of 5% CO_2_. The medium was then replaced with fresh culture medium containing DCS at final concentrations of 12.5–800 µM, and cells were incubated for a further 72 h under identical conditions.

At the end of the treatment period, the medium was aspirated and 500 µL of MTT solution (0.2 mg mL^−1^ in serum-free DMEM) was added to each well. Plates were returned to the incubator for 3 h to allow formation of insoluble formazan crystals. The MTT solution was removed, 1 mL of DMSO was added to each well, and plates were incubated for 20 min at 37 °C to solubilize the formazan.

Aliquots of the resulting colored solution (200 µL) were transferred to a 96-well plate, and absorbance was measured at 540 nm using a microplate reader. Cell viability was expressed as a percentage of the untreated control.

### 3.4. Melanin Content Assay

B16F10 cells (8 × 10^4^ cells dish^−1^) were seeded in 60 mm dishes and allowed to attach for 24 h at 37 °C in a humidified 5% CO_2_ incubator. Cells were then incubated for 72 h under the same conditions with DCS at final concentrations of 25, 50, or 100 µM. In all treatment groups, α-MSH (100 nM) was added to stimulate melanogenesis, and arbutin (300 µM) was included as a positive control.

After treatment, the culture medium was discarded and the cells were rinsed twice with ice-cold 1 × PBS. Lysis buffer (200 µL dish^−1^) consisting of RIPA buffer supplemented with 1% protease inhibitor cocktail (*v*/*v* = 100:1) was added, and the dishes were incubated on ice for 30 min. Lysates were collected with a cell scraper, transferred to 1.5 mL microtubes, and centrifuged at 15,000 rpm for 30 min at –8 °C.

The resulting pellets were resuspended in 200 µL of 1 N NaOH containing 10% (*v*/*v*) DMSO and incubated at 80 °C for 20 min to completely dissolve cellular melanin. Aliquots (200 µL) of each sample were transferred to a 96-well plate, and absorbance was measured at 405 nm using a microplate reader to quantify melanin content.

### 3.5. Intracellular Tyrosinase Activity Assay

B16F10 cells were seeded in 60 mm culture dishes at a density of 8 × 10^4^ cells dish^−1^ and pre-incubated for 24 h at 37 °C in a humidified atmosphere of 5% CO_2_. The cells were then treated for 72 h with DCS (final concentrations: 25, 50, or 100 µM) in the presence of α-MSH (100 nM) to stimulate melanogenesis; arbutin (300 µM) served as the positive control.

After treatment, the medium was discarded and the cells were washed twice with ice-cold 1 × PBS. Lysis buffer (200 µL dish^−1^; RIPA supplemented with 1% protease inhibitor cocktail, *v*/*v* = 100:1) was added, and dishes were incubated on ice for 30 min. Lysates were harvested with a cell scraper, transferred to 1.5 mL microtubes, and centrifuged at 15,000 rpm for 30 min at –8 °C (M15R, Hanil Scientific, Gimpo, Republic of Korea).

The resulting supernatants were diluted 1:10 in triple-distilled water, and total protein was quantified using a BCA protein assay kit according to the manufacturer’s instructions. For the tyrosinase reaction, 20 µL of each protein sample was mixed with 80 µL of 2 mg mL^−1^ L-DOPA prepared in 0.1 M sodium phosphate buffer (pH 6.8) in a 96-well plate. The mixture was incubated at 37 °C, and absorbance at 490 nm was recorded every 30 min for up to 90 min using a microplate reader (Epoch, BioTek, Winooski, VT, USA). Tyrosinase activity was expressed as the change in absorbance (ΔA_490_) per mg of protein over time.

### 3.6. Western Blot Analysis

B16F10 murine melanoma cells were cultured in 60 mm dishes and, after a 24 h stabilization period at 37 °C in 5% CO_2_, were treated with DCS (25, 50, or 100 µM) in the presence of the melanogenic stimulator α-MSH (100 nM); arbutin (300 µM) served as the positive control. To examine melanogenic enzymes (tyrosinase, TRP-1, and TRP-2), cells were seeded at 1.5 × 10^5^ cells dish^−1^ and incubated with the test agents for 48 h. For signaling proteins (ERK, p38, JNK, AKT, CREB, β-catenin, GSK-3β, and MITF), the cells were seeded at 3.0 × 10^5^ cells dish^−1^ and exposed for 3 h under identical culture conditions.

Following treatment, cultures were washed twice with ice-cold 1 × PBS, and 200 µL of ice-cold lysis buffer (RIPA buffer supplemented with 1% protease inhibitor cocktail, *v*/*v* = 100:1) was added to each dish. Lysates were collected with a cell scraper, transferred to microcentrifuge tubes, vortex-mixed, and clarified via centrifugation at 15,000 rpm and −8 °C for 30 min (M15R, Hanil Scientific, Gimpo, Republic of Korea). Protein concentrations in the resulting supernatants were determined with the Pierce™ BCA Protein Assay Kit, and sample concentrations were adjusted to 30 µg protein mL^−1^. Equal volumes of protein solution and 2 × Laemmli sample buffer containing 2-mercaptoethanol (Laemmli: 2-ME = 20:1, *v*/*v*) were mixed and boiled for 5 min at 100 °C; samples were stored at −20 °C until analysis.

For electrophoresis, 16 µL of each denatured sample was loaded onto SDS–polyacrylamide gels, which were run at 100 V for 30 min and then 200 V for 40 min. Proteins were transferred to PVDF membranes using the Trans-Blot Turbo Transfer System (Bio-Rad, Hercules, CA, USA). Membranes were washed four times for 5 min each in TBS containing 0.1% Tween-20 (TBS-T) and blocked for 1 h at room temperature in either 5% (*w*/*v*) skim milk (for tyrosinase, TRP-1, TRP-2, and MITF) or 5% (*w*/*v*) bovine serum albumin (for β-catenin, phospho-β-catenin, GSK-3β, phospho-GSK-3β, CREB, phospho-CREB, ERK, phospho-ERK, p38, phospho-p38, JNK, phospho-JNK, AKT, and phospho-AKT).

After six 5 min washes in TBS-T, the membranes were incubated overnight at 4 °C with the appropriate primary antibodies diluted 1:2000 in TBS-T. The next day, the membranes were washed again (6 × 5 min, TBS-T) and incubated for 90 min at room temperature with horseradish-peroxidase-conjugated anti-rabbit or anti-mouse IgG secondary antibodies diluted 1:1000 in the same buffer (5% skim milk or 5% BSA in TBS-T).

Immunoreactive bands were visualized with an enhanced chemiluminescence detection kit and captured using a FUSION Solo S imaging system (Vilber Lourmat, Collégien, France). Band intensities were quantified with ImageJ software and normalized to the corresponding β-actin loading control.

### 3.7. Protocol for Assessing Primary Skin Irritation in Humans

To determine whether the investigated substance may cause irritation on human skin, a primary irritation study was carried out in accordance with the Personal Care Products Council (PCPC, 2014) guidelines and the internal standard operating procedures of DermaPro Co., Ltd. The study protocol was reviewed and approved by the Institutional Review Board (IRB) of DermaPro and complied with the ethical standards of the Declaration of Helsinki. All participants gave written informed consent after being fully informed about the study’s objectives, procedures, and potential risks. A total of 32 healthy volunteers (31 females and 1 male), aged 19 to 60 years (mean age 46.59 ± 8.17 years), were enrolled based on pre-established inclusion and exclusion criteria. Participant eligibility was confirmed through a detailed review of their medical history and general health status. Prior to application, the designated test area (the upper back) was disinfected using 70% ethanol. A volume of 20 μL of the test material was applied under occlusion using a patch for 24 h. After removal of the patch, skin reactions were evaluated at two time points: 20 min and 24 h. Assessments were conducted by a qualified dermatologist using criteria set forth by the PCPC, focusing on signs of erythema, edema, or other adverse dermal responses. Throughout the study, participant safety was continuously monitored, and any untoward effects were recorded and managed in accordance with ethical guidelines. This investigation was implemented under strict ethical oversight and adhered to all required regulatory and scientific standards.

### 3.8. Statistical Analysis

All data are presented as mean ± standard deviation (SD) from three independent experiments. Statistical analyses were performed using one-way analysis of variance (ANOVA) with appropriate post hoc tests, using WINKS SDA Version 7.0.9 Professional (TexaSoft, Cedar Hill, TX, USA). Statistical significance is indicated as follows: * *p* < 0.05, ** *p* < 0.01, *** *p* < 0.001.

## 4. Conclusions

**Figure 7:**
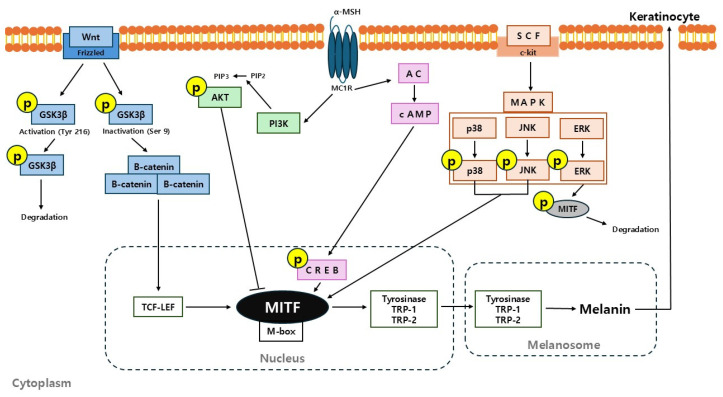
Schematic diagram of melanogenesis-related pathways modulated by DCS. The figure illustrates key signaling pathways involved in melanogenesis, including Wnt/β-catenin, PI3K/AKT, cAMP/CREB, and MAPK (p38, JNK, ERK), all converging on MITF regulation. DCS is proposed to inhibit melanogenesis by (1) reducing MITF degradation via MAPK inhibition, (2) stabilizing β-catenin through GSK3β suppression, and (3) downregulating MITF expression via inhibiting CREB phosphorylation.

In summary, this study provides compelling evidence that D-cycloserine (DCS), beyond its established antimicrobial role, exhibits potent anti-melanogenic activity by modulating multiple signaling cascades involved in melanin biosynthesis. DCS significantly downregulated the expression of key melanogenic enzymes including tyrosinase, TRP-1, and TRP-2, as well as the master transcription factor MITF, in melanoma cells. Mechanistic investigations revealed that DCS suppresses the phosphorylation of CREB, p38, and JNK, while simultaneously enhancing ERK activation and promoting β-catenin degradation via GSK-3β activation (Figure 7).

Importantly, DCS demonstrated excellent skin compatibility, being classified as non-irritating in a primary human skin irritation test, indicating strong potential for topical applications.

When compared to arbutin, a widely used commercial skin-whitening agent, DCS exhibited similar modulatory effects on the MAPK and GSK-3β/β-catenin signaling pathways. Notably, DCS exerted its depigmenting effects at a lower concentration (100 μM) than arbutin (300 μM), indicating superior efficacy. Furthermore, while arbutin poses a safety concern due to its enzymatic conversion by skin microbiota into hydroquinone—a potentially harmful compound—DCS lacks such metabolic risks due to its stable molecular structure.

These mechanistic and safety-related advantages strongly support the repositioning of DCS as a promising and effective cosmeceutical candidate for the treatment of pigmentary disorders. However, we acknowledge that the current study does not determine whether DCS modulates the MAPK and PI3K/AKT pathways directly or indirectly through upstream regulators such as CREB. Future studies incorporating pathway-specific inhibitors (e.g., ERK or PI3K blockers) will be essential to delineate the precise signaling hierarchy and specificity of DCS-mediated effects.

Moreover, while this study presents a strong theoretical foundation for drug repurposing, we recognize that transitioning DCS from systemic to topical use may present regulatory and formulation challenges. These include issues such as skin penetration, stability in topical vehicles, and re-evaluation of safety profiles under cosmetic regulations. Addressing these practical hurdles will be an essential step toward successful clinical translation.

## Figures and Tables

**Table 1:** Results of the primary skin irritation test (*n* = 32).

No	Test Sample	No. of Responses	1st Assessment				2nd Assessment				Reaction Grade (R) *
			+1	+2	+3	+4	+1	+2	+3	+4	
1	D-cycloserine (50 μM)	0	0	0	0	0	0	0	0	0	0
2	D-cycloserine (100 μM)	0	0	0	0	0	0	0	0	0	0

* None to slight: 0.00 ≤ R < 0.87.

## Data Availability

The authors confirm that all the data needed to support this study are presented within the article.

## References

[1] Akl J., Lee S., Ju H.J., Parisi R., Kim J.Y., Jeon J.J., Heo Y.W., Eleftheriadou V., Hamzavi I., Griffiths C.E.M. (2024). Global Vitiligo Atlas. Estimating the burden of vitiligo: A systematic review and modelling study. Lancet Public Health.

[2] Ismail I.B., Bhat Y.J., Ul Islam M.S. (2025). Treatment Advances in Vitiligo: An Updated Review. Dermatol. Pract. Concept..

[3] Dabas G., Vinay K., Parsad D., Kumar A., Kumaran M.S. (2020). Psychological disturbances in patients with pigmentary disorders: A cross-sectional study. J. Eur. Acad. Dermatol. Venereol..

[4] Pinzi L., Bisi N., Rastelli G. (2024). How drug repurposing can advance drug discovery: Challenges and opportunities. Front. Drug Discov..

[5] Beninger P. (2025). Thalidomide: Following Tragedy, a Repurposed Molecule With Continuing Opportunities for Clinical Benefit. Clin. Ther..

[6] Ala M., Mohammad Jafari R., Dehpour A.R. (2021). Sildenafil beyond erectile dysfunction and pulmonary arterial hypertension: Thinking about new indications. Fundam. Clin. Pharmacol..

[7] Banerjee S., Banerjee D., Singh A., Kumar S., Pooja D., Ram V., Kulhari H., Saharan V.A. (2023). A Clinical Insight on New Discovered Molecules and Repurposed Drugs for the Treatment of COVID-19. Vaccines.

[8] Matsuo H., Kumagai T., Mori K., Sugiyama M. (2003). Molecular cloning of a D-cycloserine resistance gene from D-cycloserine-producing *Streptomyces garyphalus*. J. Antibiot..

[9] Kumagai T., Koyama Y., Oda K., Noda M., Matoba Y., Sugiyama M. (2010). Molecular cloning and heterologous expression of a biosynthetic gene cluster for the antitubercular agent D-cycloserine produced by *Streptomyces lavendulae*. Antimicrob. Agents Chemother..

[10] Pendela M., Dragovic S., Bockx L., Hoogmartens J., Van Schepdael A., Adams E. (2008). Development of a liquid chromatographic method for the determination of related substances and assay of d-cycloserine. J. Pharm. Biomed. Anal..

[11] Zhuang J., Yang W., Cheng G.J. (2025). Mechanistic Insights into the Reversible Inhibition of d-Cycloserine in Alanine Racemase from *Mycobacterium tuberculosis*. J. Chem. Inf. Model..

[12] Otto M.W., Kredlow M.A., Smits J.A.J., Hofmann S.G., Tolin D.F., de Kleine R.A., van Minnen A., Evins A.E., Pollack M.H. (2016). Enhancement of Psychosocial Treatment With D-Cycloserine: Models, Moderators, and Future Directions. Biol. Psychiatry.

[13] Ni J., Wang N., Gao L., Li L., Zheng S., Liu Y., Ozukum M., Nikiforova A., Zhao G., Song Z. (2016). The effect of the NMDA receptor-dependent signaling pathway on cell morphology and melanosome transfer in melanocytes. J. Dermatol. Sci..

[14] Zhang H., Chen Z., Zhang A., Gupte A.A., Hamilton D.J. (2022). The Role of Calcium Signaling in Melanoma. Int. J. Mol. Sci..

[15] Liu Y., Jia Y., Kou Z. (2025). Bibliometric analysis of NMDA receptors: 2015–2024. Front. Pharmacol..

[16] Berns H.M., Watkins-Chow D.E., Lu S., Louphrasitthiphol P., Zhang T., Brown K.M., Moura-Alves P., Goding C.R., Pavan W.J. (2024). Single-cell profiling of MC1R-inhibited melanocytes. Pigment Cell Melanoma Res..

[17] Kang H.K., Hyun C.G. (2020). Anti-inflammatory effect of d-(+)-cycloserine through inhibition of NF-κB and MAPK signaling pathways in LPS-induced RAW 264.7 macrophages. Nat. Prod. Commun..

[18] King S., Campbell J., Rowe R., Daly M.L., Moncrieff G., Maybury C. (2023). A systematic review to evaluate the efficacy of azelaic acid in the management of acne, rosacea, melasma and skin aging. J. Cosmet. Dermatol..

[19] Sauer N., Oślizło M., Brzostek M., Wolska J., Lubaszka K., Karłowicz-Bodalska K. (2023). The multiple uses of azelaic acid in dermatology: Mechanism of action, preparations, and potential therapeutic applications. Postepy Dermatol. Alergol..

[20] Konisky H., Balazic E., Jaller J.A., Khanna U., Kobets K. (2023). Tranexamic acid in melasma: A focused review on drug administration routes. J. Cosmet. Dermatol..

[21] Zhang J., Gu D., Yan Y., Pan R., Zhong H., Zhang C., Xu Y. (2024). Potential Role of Tranexamic Acid in Rosacea Treatment: Conquering Flushing Beyond Melasma. Clin. Cosmet. Investig. Dermatol..

[22] Cho Y.S., Kim H.O., Woo S.M., Lee D.H. (2022). Use of Dexpanthenol for Atopic Dermatitis-Benefits and Recommendations Based on Current Evidence. J. Clin. Med..

[23] Gorski J., Proksch E., Baron J.M., Schmid D., Zhang L. (2020). Dexpanthenol in Wound Healing after Medical and Cosmetic Interventions (Postprocedure Wound Healing). Pharmaceuticals.

[24] Han H.J., Hyun C.G. (2023). Acenocoumarol exerts anti-inflammatory activity via the suppression of NF-κB and MAPK pathways in RAW 264.7 cells. Molecules.

[25] Kim H.M., Hyun C.G. (2022). Miglitol, an oral antidiabetic drug, downregulates melanogenesis in B16F10 melanoma cells through the PKA, MAPK, and GSK3β/β-Catenin signaling pathways. Molecules.

[26] Kim T., Hyun C.G. (2022). Imperatorin positively regulates melanogenesis through signaling pathways involving PKA/CREB, ERK, AKT, and GSK3β/β-catenin. Molecules.

[27] Moon S.H., Chung Y.C., Hyun C.G. (2019). Tobramycin promotes melanogenesis by upregulating p38 MAPK protein phosphorylation in B16F10 melanoma cells. Antibiotics.

[28] Wang F., Ma W., Fan D., Hu J., An X., Wang Z. (2024). The biochemistry of melanogenesis: An insight into the function and mechanism of melanogenesis-related proteins. Front. Mol. Biosci..

[29] Yoon J.H., Youn K., Jun M. (2022). Discovery of Pinostrobin as a Melanogenic Agent in cAMP/PKA and p38 MAPK Signaling Pathway. Nutrients.

[30] Huang H.C., Yen H., Lu J.Y., Chang T.M., Hii C.H. (2020). Theophylline enhances melanogenesis in B16F10 murine melanoma cells through the activation of the MEK 1/2, and Wnt/β-catenin signaling pathways. Food Chem. Toxicol..

[31] Cho J., Bejaoui M., Tominaga K., Isoda H. (2024). Comparative Analysis of Olive-Derived Phenolic Compounds’ Pro-Melanogenesis Effects on B16F10 Cells and Epidermal Human Melanocytes. Int. J. Mol. Sci..

[32] Lee Y.J., Hyun C.G. (2024). Mechanistic Insights into the Stimulatory Effect of Melanogenesis of 4-Methylcoumarin Derivatives in B16F10 Melanoma Cells. Int. J. Mol. Sci..

[33] Hassan M., Shahzadi S., Kloczkowski A. (2023). Tyrosinase Inhibitors Naturally Present in Plants and Synthetic Modifications of These Natural Products as Anti-Melanogenic Agents: A Review. Molecules.

[34] Hirobe T. (2024). Role of Dermal Factors Involved in Regulating the Melanin and Melanogenesis of Mammalian Melanocytes in Normal and Abnormal Skin. Int. J. Mol. Sci..

[35] Zolghadri S., Beygi M., Mohammad T.F., Alijanianzadeh M., Pillaiyar T., Garcia-Molina P., Garcia-Canovas F., Munoz-Munoz J., Saboury A.A. (2023). Targeting tyrosinase in hyperpigmentation: Current status, limitations and future promises. Biochem. Pharmacol..

[36] Bin B.H., Kim S.T., Bhin J., Lee T.R., Cho E.G. (2016). The Development of Sugar-Based Anti-Melanogenic Agents. Int. J. Mol. Sci..

[37] Tanemura A., Yang L., Yang F., Nagata Y., Wataya-Kaneda M., Fukai K., Tsuruta D., Ohe R., Yamakawa M., Suzuki T. (2015). An immune pathological and ultrastructural skin analysis for rhododenol-induced leukoderma patients. J. Dermatol. Sci..

[38] Imokawa G., Ishida K. (2014). Inhibitors of intracellular signaling pathways that lead to stimulated epidermal pigmentation: Perspective of anti-pigmenting agents. Int. J. Mol. Sci..

[39] Lin X., Meng X., Lin J. (2023). The possible role of Wnt/β-catenin signalling in vitiligo treatment. J. Eur. Acad. Dermatol. Venereol..

[40] Bai R., Guo Y., Liu W., Song Y., Yu Z., Ma X. (2023). The Roles of WNT Signaling Pathways in Skin Development and Mechanical-Stretch-Induced Skin Regeneration. Biomolecules.

[41] Qian W., Liu W., Zhu D., Cao Y., Tang A., Gong G., Su H. (2020). Natural skin-whitening compounds for the treatment of melanogenesis (Review). Exp. Ther. Med..

[42] Zhang Y., Wang S., Yang A. (2025). Hydrolyzed conchiolin protein inhibits melanogenesis through PKA/CREB and MEK/ERK signalling pathways. Int. J. Cosmet. Sci..

[43] Liu C., Nueraihemaiti M., Zang D., Edirs S., Zou G., Aisa H.A. (2023). Quercetin 3-O-(6″-O-E-caffeoyl)-β-D-glucopyranoside, a Flavonoid Compound, Promotes Melanogenesis through the Upregulation of MAPKs and Akt/GSK3β/β-Catenin Signaling Pathways. Int, J, Mol, Sci..

[44] Liu B., Xie Y., Wu Z. (2020). Astragaloside IV Enhances Melanogenesis via the AhR-Dependent AKT/GSK-3β/β-Catenin Pathway in Normal Human Epidermal Melanocytes. Evid.-Based Complement. Altern. Med..

[45] Ouyang J., Hu N., Wang H. (2024). Petanin Potentiated JNK Phosphorylation to Negatively Regulate the ERK/CREB/MITF Signaling Pathway for Anti-Melanogenesis in Zebrafish. Int. J. Mol. Sci..

[46] Liu J., Xu X., Zhou J., Sun G., Li Z., Zhai L., Wang J., Ma R., Zhao D., Jiang R. (2023). Phenolic acids in Panax ginseng inhibit melanin production through bidirectional regulation of melanin synthase transcription via different signaling pathways. J. Ginseng Res..

[47] Li J., Zou W., Li M., Yan Y., Yu Y., Li X., Ma Y.L. (2025). Aloe Vera flowers extracts inhibit melanogenesis via activating PI3K/Akt signaling pathway: Network pharmacology and experimental validation. Fitoterapia.

[48] Zhou S., Zeng H., Huang J., Lei L., Tong X., Li S., Zhou Y., Guo H., Khan M., Luo L. (2021). Epigenetic regulation of melanogenesis. Ageing Res. Rev..

[49] Zhou Y., Zeng H.L., Wen X.Y., Jiang L., Fu C.H., Hu Y.B., Lei X.X., Zhang L., Yu X., Yang S.Y. (2022). Selaginellin Inhibits Melanogenesis via the MAPK Signaling Pathway. J. Nat. Prod..

[50] Wusiman Z., Zhang A.M., Zhang S.S., Zhao P.P., Kang Y.T., Zhang Y., Li Z.J., Huo S.X. (2025). Galangin ameliorates PTU-induced vitiligo in zebrafish and B16F10 cells by increasing melanogenesis through activation of the p38/JNK MAPK pathway. Front. Pharmacol..

[51] Li X., Chen L., Wang H., Li Y., Wu H., Guo F. (2024). Germacrone, isolated from Curcuma wenyujin, inhibits melanin synthesis through the regulation of the MAPK signaling pathway. J. Nat. Med..

[52] Kim S.H., Lee J., Jung J., Kim G.H., Yun C.Y., Jung S.H., Hwang B.Y., Hong J.T., Han S.B., Jung J.K. (2024). Interruption of p38MAPK-MSK1-CREB-MITF-M pathway to prevent hyperpigmentation in the skin. Int. J. Biol. Sci..

[53] Chen H., Jian M., Teng H., Li Z., Xu X., Li X., Jiang R., Zhao D., Sun L., Liu J. (2025). Ginsenoside Rf in wild ginseng adventitious roots extract inhibits melanogenesis via cAMP/PKA and NO/cGMP signalling pathways in α-melanocyte-stimulating hormone-stimulated B16F10 mouse melanoma cells and zebrafish. Nat. Prod. Res..

[54] An X., Lv J., Wang F. (2022). Pterostilbene inhibits melanogenesis, melanocyte dendricity and melanosome transport through cAMP/PKA/CREB pathway. Eur. J. Pharmacol..

[55] Cao Y., Lv J., Tan Y., Chen R., Jiang X., Meng D., Zou K., Pan M., Tang L. (2024). Tribuloside acts on the PDE/cAMP/PKA pathway to enhance melanogenesis, melanocyte dendricity and melanosome transport. J. Ethnopharmacol..

